# The Congress of American Physicians and Surgeons

**Published:** 1888-10

**Authors:** 


### *THE CONGRESS OF AMERICAN PHYSICIANS AND SURGEONS.*

It is something like three years since it was first proposed by Dr. C. H. Mastin, of Mobile, Alabama, to organize a medical congress which should have for its constituent parts the several American special societies or associations. The proposition was so novel that it struck with something like the impact of a thunderbolt, even in the society where it was first proposed. Some warmly supported Dr. Mastin from almost the very outset; others held an armed neutrality, so to speak, while yet others openly and strongly opposed the scheme. However, as time went on, and it began to take definite shape through the action of the various organizations which were particularly interested, those who doubted the propriety of the project, or were at most occupying neutral ground, finally became some of its most active promoters, if not, indeed, high officials in the councils of the Congress.

The year 1888 witnessed the complete organization and final consummation of the plans of this medical body, which, under proper guidance, is destined to become, probably, the most famous medical organization in the world.

The work done at this first triennial session has already been recorded and passed into history. The medical journals throughout the land have, for the most part, yielded a very cordial support to its purposes and aided by their kindly words the accomplishment of its objects.

For our part, we can only say that, of the more than two hundred papers on scientific subjects presented to that body, all were fairly good, some were most excellent, and a few will mark an epoch in the literature of American medicine.

A noticeable feature of the Congress was a departure from the ordinary methods of social entertainment, as well as an absence of the ordinary surgical and pharmaceutical displays. It remains to be seen whether these are improvements or not.

Coming to the business part of the matter, the Executive Committee is to be commended for the simplicity of its rules and for the unanimity which has prevailed in its councils.

A few of the special societies have not yet been admitted, by reason of their youth, a resolution of the Executive Committee providing that no society having had less than two years' existence shall be admitted to membership. The doubtful propriety of passing such an *ex post facto* resolution becomes apparent when societies with such men as Dr. Abraham Jacobi, the distinguished president of the New York Academy of Medicine, and Dr. Joseph Leidy, the eminent anatomist, at their head, are denied participation with the other special organizations. However, time will serve to correct all such irregularities, and we may confidently predict that the Congress of 1891 is already an assured success, whether viewed from a scientific, social, or business standpoint.

